# Awareness of Cytomegalovirus Infection among Pregnant Women in Geneva, Switzerland: A Cross-sectional Study

**DOI:** 10.3390/ijerph121214982

**Published:** 2015-12-02

**Authors:** Alexia Willame, Geraldine Blanchard-Rohner, Christophe Combescure, Olivier Irion, Klara Posfay-Barbe, Begoña Martinez de Tejada

**Affiliations:** 1 Department of Obstetrics and Gynecology, University of Geneva Hospitals and Faculty of Medicine, 30 Boulevard de la Cluse, 1211 Geneva 14, Switzerland; Alexia.Willame@hcuge.ch (A.W.); Olivier.Irion@hcuge.ch (O.I.); 2 Department of Pediatrics, Children’s Hospital, University of Geneva Hospitals, 6 Rue Willy-Donzé, 1211 Geneva 14, Switzerland; Geraldine.BlanchardRohner@hcuge.ch (G.B.-R.); Klara.PosfayBarbe@hcuge.ch (K.P.-B.); 3 Clinical Research Center, University of Geneva Hospitals, 4 Rue Gabrielle Perret-Gentil, 1211 Geneva 14, Switzerland; Christophe.Combescure@hcuge.ch

**Keywords:** cytomegalovirus, pregnancy, information, awareness, infection, congenital, knowledge

## Abstract

*Background***:** Cytomegalovirus (CMV) is the most frequent cause of congenital infection and commonly associated with sensorineural deficit. At present, there is neither prophylaxis nor treatment during pregnancy. The objective of this study was to evaluate the level of awareness regarding CMV infection and its consequences in women delivering at the University of Geneva Hospitals (Geneva, Switzerland). *Methods*: The study consisted of a validated questionnaire completed by women in the immediate postpartum period. *Results*: The questionnaire was completed by 59% (314/528) of delivering women. Only 39% (123/314) knew about CMV and 19.7% (62/314) had received information about preventive measures. Women were more aware about other congenital diseases, such as toxoplasmosis (87%); human immunodeficiency virus (99%); syphilis (85.5%); rubella (92.3%); and group B *Streptococcus* (63%). Factors associated with CMV awareness were Swiss nationality, high education level, employment in health care or with children, and being followed by an obstetrician. Regarding quality of information, few were aware of the main CMV complications (deafness, 25.2%; mental retardation, 34.5%). Among those informed about CMV, most (74.6%) knew about preventive measures. Among these, 82.5% thought that these were easily applicable. *Conclusions*: Most women are unaware of CMV infection and its potential risks during pregnancy. It is crucial to improve CMV information given to pregnant women to prevent the risks for the fetus/newborn.

## 1. Introduction

Cytomegalovirus is the most common congenital infection and it is recognized as the most frequent cause of neurological handicap of infectious origin since universal rubella vaccination was implemented [1]. Congenital infection is detected in 0.6%–4% newborns, depending on the studied population [2,3,4,5,6]. In industrialized countries, 40%–50% women of childbearing age are CMV-seronegative, but an average of 0.5%–1% pregnant women will seroconvert per year [1,5,7]. Primary infection leads to transmission rates of 30%–40% and secondary infection, such as reactivation or reinfection by a new strain, can also infect the fetus, but with lower rates of 0.5%–2% [3,5,7,8,9,10]. Approximately 10% of infected fetuses will develop severe complications, such as microcephaly, growth retardation, hepatosplenomegalia, or death. Among the 90% asymptomatic infected newborns, 10%–15% will develop hearing loss or mental retardation in the long term [2,7,8].

At present, there is no validated treatment for CMV infection during pregnancy. Various prospective and retrospective studies showed encouraging results for the effectiveness of intravenous hyperimmune globulin to prevent or treat CMV congenital disease [11,12,13,14,15,16,17,18,19,20]. However, a recent randomized trial including 123 women (61 in the gammaglobulin arm and 62 in the placebo arm) did not show a statistically significant benefit of gammaglobulin (30% congenital transmission rate in the hypergammaglobulin group *vs.* 44% in the placebo group; *p* < 0.13) [21]. Regarding infants with symptomatic congenital CMV, treatment with valgancyclovir showed promising results and seemed to be the safest among different antiviral drugs [22,23,24,25,26,27,28,29,30].

Vaccination is difficult as even secondary infection can lead to CMV fetal infection [31,32,33,34,35,36]. Due to lack of treatment and the partial rate of vertical transmission, there is currently no recommendation for CMV screening during pregnancy even though 7/10,000 newborns are infected by CMV and are symptomatic [37,38]. Of note, this is almost twice the 4.3/10,000 infected by group B *Streptococcus* for which screening is systematic [1]. To date, hygiene measures are the only recommended intervention to prevent CMV infection [39,40,41,42,43]. The United States Centers for Disease Control and Prevention recommend avoiding contact with human body fluids, such as urine, saliva, tears, feces, or semen [44]. Women in contact with children less than 4-years-old are a particularly high-risk group [6,45,46] and are 10 times more likely to seroconvert than other groups [45]. Applying recommended hygiene measures would allow an 80% reduction of seroconversion [41,43]. However, pregnant women have poor knowledge of CMV infection compared to other congenital diseases, such as Down syndrome, toxoplasmosis, and human immunodeficiency virus (HIV) infection [47,48,49]. Studies have shown also that less than 50% of obstetricians provide pregnant women with information about CMV and preventive measures [50,51,52].

The aim of the present study was to evaluate the knowledge of CMV infection and prevention among pregnant women in Geneva, Switzerland, together with factors associated with being well informed, including a comparison with knowledge of other congenital diseases. Similar to other studies, our results would confirm that healthcare professionals need to heighten awareness of the risks of CMV infection in this patient population.

## 2. Experimental Section

### 2.1. Participants and Study Design

This single-center, cross-sectional study was conducted at the postpartum unit of the maternity division of the University of Geneva Hospitals (Geneva, Switzerland), a referral center with approximately 4000 deliveries per year. Most women were followed by private obstetricians and referred to the hospital at around 36 weeks of gestation to continue pregnancy follow-up through to delivery and postpartum. We aimed to recruit around 300 women. Taking into account that some women would decline participation, we estimated that we would be able to complete recruitment within two months.

All women who delivered in April and May 2013 and stayed in the postpartum unit for at least 24 h received a study questionnaire. No exclusion criteria were applied and willingness to complete the questionnaire was the only requirement for study participation. The questionnaire was distributed to all newly hospitalized women on a daily basis. Women were orally informed about the study by midwives. Those accepting to participate were requested to sign a consent form and to return the questionnaire the next day to one of the recruiting staff or to drop it off in an anonymous box. A list of hospitalized women to whom the questionnaire was given was recorded daily. The study was approved by the ethics committee of the University of Geneva Hospitals.

### 2.2. Questionnaire

The questionnaire was adapted from a previous questionnaire established and validated by a French group [40,53] and consisted of 49 questions grouped into four sections (see Supplementary File). Section 1 intended to evaluate the knowledge of general congenital problems. Section 2 contained questions about CMV and preventive measures. Section 3 consisted of questions on pregnancy follow-up. Section 4 contained sociodemographic questions, such as age, origin, and education level. The French questionnaire was translated into English, Spanish, Portuguese and German, the most frequently spoken languages in our area. The database was created using a specific software (Cardiff TeleForm, version 10.2, Cardiff, Vista, CA, USA) that transfers the data of a scanned questionnaire directly into a database.

### 2.3. Statistical Analysis

Factors were described by counts and percentage. Participants’ characteristics were compared between women with and without knowledge of CMV using chi-squared tests or Fisher’s exact test. Odds ratios were also reported. Multivariable logistic regression analysis was conducted to assess independent associations between these characteristics and knowledge of CMV. The association between the knowledge of CMV and the practitioners who followed the women during the 1st trimester was assessed with an additional logistic regression model in the subgroup of women with a follow-up during the 1st trimester. Sources of information regarding CMV and preventive measures were compared according to the type of follow-up (midwife, obstetrician or general practitioner) using Fisher’s exact test. The proportions of women aware of CMV and other congenital diseases were compared using the McNemar test. Statistical analyses were performed using S-plus 8.0 for Windows (Insightful Corp, Seattle, WA, USA). All statistical tests were two-sided and a *p*-value < 0.05 was considered as statistically significant.

## 3. Results

### 3.1. Participants

**Figure 1:**
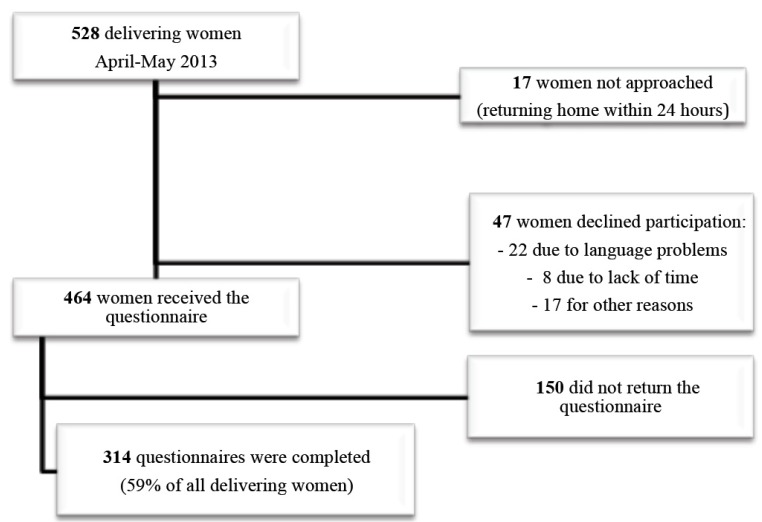
Flowchart of participants’ enrollment.

During the study period, 528 women delivered at our maternity unit and a total of 314 questionnaires (59% participation rate) were collected (Figure 1). Sociodemographic characteristics of participants are shown in Table 1, together with a comparison between women with and without knowledge of CMV infection. The largest group of respondents was women of Swiss nationality (40.5%) who answered in French. Mean age of respondents was 32.2 ± 5.3 years (range, 16–45 years). The majority of pregnant women had been followed by an obstetrician (78.6%) and the remaining ones by either a general practitioner (10.5%) or a midwife (10.9%).

**Table 1:** Sociodemographic characteristics of participants.

			Knowledge of CMV		Odds Ratio (95% CI)			
		All (*n* = 314)	No (*n* = 191)	Yes (*n* = 123)	Univariate	*p*-value	Adjusted *	*p*-value
*Nationality*	*Swiss*	125/309 (40.5%)	61/187 (32.6%)	64/121 (52.5%)	1 (reference)	0.002	1 (reference)	0.004
	*European*	102/309 (33.3%)	69/187 (36.9%)	33/121 (27.0%)	0.5 (0.3;0.8)		0.4 (0.2;0.7)	0.003
	*Other*	82/309 (26.5%)	57/187 (30.5%)	25/121 (20.5%)	0.4 (0.2;0.8)		0.4 (0.2;0.8)	0.008
*Language*	*French, German*	269/314 (85.7%)	160/191 (83.8%)	109/123 (88.6%)	1 (reference)	0.12		
	*English*	30/314 (9.6%)	18/191 (9.4%)	12/123 (9.8%)	1.0 (0.5;2.1)			
	*Spanish, Portuguese*	15/314 (4.8%)	13/191 (6.8%)	2/123 (1.6%)	0.2 (0.0;1.0)			
*Age*	*≤25 years*	34/311 (10.9%)	25/188 (13.3%)	9/123 (7.3%)	1 (reference)	0.25	1 (reference)	0.38
	*26 to 35 years*	192/311 (61.7%)	112/188 (59.6%)	80/123 (65.0%)	2.0 (0.9;4.5)		1.4 (0.5;3.8)	0.49
	*≥36 years*	85/311 (27.3%)	51/188 (27.1%)	34/123 (27.6%)	1.9 (0.8;4.5)		1.0 (0.3;2.8)	0.93
*Education level*	*Minimal schooling*	43/306 (14.1%)	37/184 (20.1%)	6/122 (4.9%)	1 (reference)	<0.001	1 (reference)	<0.001
	*Apprenticeship*	53/306 (17.3%)	38/184 (20.7%)	15/122 (12.3%)	2.4 (0.9;7.0)		1.2 (0.4;4.0)	0.74
	*High school*	74/306 (24.2%)	44/184 (23.9%)	30/122 (24.6%)	4.2 (1.6;11.2)		2.9 (1.0;8.5)	0.06
	*University*	136/306 (44.4%)	65/184 (35.3%)	71/122 (58.2%)	6.7 (2.7;17.0)		6.0 (2.2;16.4)	<0.001
*Employment*	*Not exposed to risk*	263/304 (86.5%)	172/184 (93.5%)	91/120 (75.8%)	1 (reference)	<0.001	1 (reference)	<0.001
	*Healthcare-related*	25/304 (8.2%)	6/184 (3.3%)	19/120 (15.8%)	6.0 (2.3;15.5)		6.9 (2.4;19.4)	<0.001
	*With children*	16/304 (5.3%)	6/184 (3.3%)	10/120 (8.3%)	3.2 (1.1;8.9)		5.7 (1.6;20.0)	0.006
*Parity*	*Primiparity*	129/308 (41.9%)	83/185 (44.9%)	46/123 (37.4%)	1 (reference)	0.24	1 (reference)	
	*Multiparity*	179/308 (58.1%)	102/185 (55.1%)	77/123 (62.6%)	1.4 (0.9;2.2)		1.5 (0.9;2.6)	0.13
*Follow-up during 1st*	*No*	8/312 (2.6%)	5/189 (2.6%)	3/123 (2.4%)	1 (reference)	1		
*trimester of pregnancy*	*Yes*	304/312 (97.4%)	184/189 (97.4%)	120/123 (97.6%)	1.0 (0.3;3.6)			
*Pregnancy follow-up by*	*General practitioner*	31/294 (10.5%)	22/177 (12.4%)	9/117 (7.7%)	1 (reference)	0.01		
	*Midwife*	32/294 (10.9%)	26/177 (14.7%)	6/117 (5.1%)	0.6 (0.2;1.8)			
	*Obstetrician*	231/294 (78.6%)	129/177 (72.9%)	102/117 (87.2%)	1.9 (0.9;4.4)			

* adjusted for multivariable analysis.

### 3.2. Knowledge of Congenital Diseases

Among the 314 respondents, 123/314 (39%) had knowledge of CMV. By comparison, 66/302 (21.9%; *p* < 0.001) knew about parvovirus B19; 286/310 (92.3%; *p* < 0.001) about rubella; 268/309 (87%; *p* < 0.001) about toxoplasmosis; 194/308 (63%; *p* < 0.001) about group B *Streptococcus*; 265/310 (85.5%; *p* < 0.001) about syphilis; 299/312 (95.8%; *p* < 0.001) about hepatitis B; 309/314 (99%, *p* < 0.001) about HIV; 187/310 (60.3%; *p* < 0.001) about Down syndrome; 170/308 (55.2%; *p* < 0.001) about fetal-alcoholic syndrome; 264/312 (84.6%; *p* < 0.001) about sudden death syndrome; 277/310 (89.4%; *p* < 0.001) about autism; and 149/306 (48.7%; *p* = 0.005) about spina bifida.

### 3.3. General Knowledge of CMV and Associated Factors

Only 62/314 (19.7%) respondents had received information about CMV preventive measures during pregnancy. Factors associated with knowledge of CMV were Swiss nationality, high education level (university degree or equivalent), working in healthcare or with children, and being followed by an obstetrician (Table 1). These factors were also associated in the multivariable analysis (Table 1). Evaluation of the type of follow-up during the 1st trimester showed that women were more frequently aware about CMV if they were followed by an obstetrician than by a midwife or a general practitioner. However, after adjustment on nationality, age, education level, and type of employment, this association was no longer statistically significant (*p* = 0.10) (Table 1).

Sources of information about CMV infection based on the type of follow-up (general practitioner, midwife, or obstetrician) are described in Table 2. Among women followed by an obstetrician, 102/231 (44.2%) knew about CMV. Of these, the majority (71.3%) had been specifically informed by the obstetrician. Fewer women followed by general practitioners (29%) or midwives (18.8%) knew about CMV infection and their sources of information were more heterogeneous (pediatrician, media, friends and family). Moreover, among women knowing about CMV, only half had been actively informed about preventive measures during pregnancy. Again, physicians were often the source of information of preventive measures.

### 3.4. Quality of Knowledge about CMV Infection and Preventive Measures

Table 3 shows specific knowledge about the consequences of CMV infection during pregnancy among women aware of CMV (*n* = 123). More than 30% of women (34.5%; 40/116) did not know if CMV infection was dangerous for the fetus. Only 37/110 (33.65%) answered correctly to more than three questions. The most common symptoms of congenital CMV infection (deafness (30/119; 25.2%) and mental retardation (41/119; 34.5%)) were rarely known.

**Table 2:** Sources of information about CMV and preventive measures based on type of first trimester follow-up.

**(A)**	**Women Followed-Up in the First Trimester by**				
	GP * (*n* = 31)	**Midwife (*n* = 32)**	**Obstetrician (*n* = 231)**	**Total (*n* = 314)**	***p*-Value**
*Patients aware of CMV*	9 (29.0%)	6 (18.8%)	102 (44.2%)	123 (39.2%)	0.009
Source of information **					
*General practitioner*	3 (33.3%)	2 (33.3%)	5 (5.0%)	11 (9.0%)	0.003
*Obstetrician*	3 (33.3%)	2 (33.3%)	72 (71.3%)	78 (63.9%)	0.01
*Midwife*	2 (22.2%)	3 (50.0%)	8 (7.9%)	15 (12.3%)	0.006
*Pediatrician*	1 (11.1%)	1 (16.7%)	4 (4.0%)	6 (4.9%)	0.17
*Media*	0 (0.0%)	1 (16.7%)	25 (24.8%)	28 (23.0%)	0.27
*Family/friends*	1 (11.1%)	1 (16.7%)	15 (14.9%)	19 (15.6%)	1
*Missing data*	0	0	1	1	
**(B)**	**Women aware of CMV and Followed-Up in the First Trimester by**				
	GP * (*n* = 9)	**Midwife (*n* = 6)**	**Obstetrician (*n* = 102)**	**Total (*n* = 123)**	***p*-Value**
*Information about CMV preventive measures*	4 (44.2%)	2 (33.3%)	54 (52.9%)	62 (50.4%)	0.59
Source of information **					
*General practitioner*	4 (100.0%)	1 (50.0%)	1 (1.9%)	6 (9.8%)	<0.001
*Obstetrician*	0 (0.0%)	0 (0.0%)	51 (96.2%)	52 (85.2%)	<0.001
*Midwife*	0 (0.0%)	1 (50.0%)	3 (5.7%)	5 (8.2%)	0.15
*Pediatrician*	0 (0.0%)	0 (0.0%)	2 (3.8%)	2 (3.3%)	1
*Studies*	0 (0.0%)	0 (0.0%)	5 (9.4%)	5 (8.2%)	1
*Media*	0 (0.0%)	0 (0.0%)	2 (3.8%)	2 (3.3%)	1
*Family/friends*	0 (0.0%)	0 (0.0%)	7 (13.2%)	7 (11.5%)	1
*Missing data*	0	0	1	1	

* GP: General practitioner; ** Not exclusive answers, each patient can answer to several proposals; (A): Source of information about CMV infection based on the type of 1st trimester follow-up; (B): Source of information about preventive measures among women aware of CMV based on the type of 1st trimester follow-up.

**Table 3:** Specific knowledge about CMV symptoms among women who have heard of CMV.

Which Symptoms are Related to CMV?	Answers * (*n* = 123)		
	No	Yes	Do Not Know
Is CMV contagious?	2 (1.6%)	**92 (74.8%)**	29 (23.6%)
Is CMV not dangerous?	**66 (56.9%)**	10 (8.6%)	40 (34.5%)
Does CMV cause deafness?	9 (7.6%)	**30 (25.2%)**	80 (67.2%)
Does CMV cause mental retardation?	5 (4.2%)	**41 (34.5%)**	73 (61.3%)
Does CMV cause jaundice?	**36 (30.5%)**	9 (7.6%)	73 (61.9%)
Does CMV cause convulsion?	**15 (12.8%)**	15 (12.8%)	87 (74.4%)
Does CMV cause microcephaly?	18 (15.1%)	**16 (13.4%)**	85 (71.4%)
Does CMV cause cardiac malformation?	**12 (10.3%)**	22 (18.8%)	83 (70.9%)
Does CMV cause death?	10 (8.5%)	**30 (25.4%)**	78 (66.1%)
**Number of Correct Answers**			
0 to 3 correct answers	70 (63.65%)		
4 to 6 correct answers	37 (33.65%)		
7 or more correct answers	3 (2.7%)		

* Correct answers are in bold

Regarding preventive measures, 88/118 (74.6%) women answered correctly to more than five questions with a significant difference between women actively informed about preventive measures and those who were not (53/62 = 85.5% *vs.* 35/56 = 62.5% respectively; *p* < 0.007). These data are reported in Table 4. Handwashing, avoiding saliva-sharing behavior with children, e.g., through shared utensils, and avoiding kissing on the mouth were estimated as easily/very easily applicable by 98/101 (97%), 88/97 (90.7%), and 84/98 (84.9%) women, respectively. Surprisingly, avoiding contact with urine was estimated as difficult to apply by 54/94 (57.4%) women. In comparison, recommended preventive measures for toxoplasmosis infection, such as avoiding eating raw meat, were evaluated as easily/very easily applicable by only 49/65 (75.4%) women.

**Table 4:** Specific knowledge about CMV preventive measures among women who have heard of CMV.

Which Hygiene Measure Prevents CMV?	Answers * (*n* = 123)		
	No	Yes	Do Not Know
Handwashing	6 (5.0%)	**88 (72.7%)**	27 (22.3%)
Not sharing the same tool	12 (9.9%)	**81 (66.95%)**	28 (23.15%)
Using mosquito repellent	**68 (56.2%)**	2 (1.7%)	51 (42.1%)
Avoiding eating raw meat/dairy products	**80 (66.1%)**	17 (14.05%)	24 (19.8%)
Drinking caffeinated drinks	**97 (80.2%)**	1 (0.8%)	23 (19.0%)
Avoiding cleaning the cat litter box	**69 (57.5%)**	30 (25.0%)	21 (17.5%)
Avoiding contact with urine	12 (10.1%)	**80 (67.2%)**	27 (22.7%)
Exercising	**88 (72.7%)**	7 (5.8%)	26 (21.5%)
Avoiding kissing on the mouth	11.9 (9.2%)	84 (70.6%)	24 (20.2%)
**Number of Correct Answers**			
0 to 1 correct answer	16 (13.55%)		
2 to 4 correct answers	14 (11.85%)		
5 or more correct answers	88 (74.6%)		

* Correct answers are in bold

## 4. Discussion

Our study revealed that few women delivering in Geneva were aware of CMV infection and even less were actively informed about CMV preventive measures during pregnancy, regardless of the fact that most women had been followed since the 1st trimester of pregnancy. Knowledge of CMV infection was much lower than that of other infections, such as HIV, rubella, syphilis, and toxoplasmosis, even though their incidence is much lower during pregnancy. In addition, the quality of knowledge about CMV was poor.

To our knowledge, this is the first study to evaluate awareness of CMV in Switzerland compared to 12 other congenital diseases. In addition, it is also the first to reveal that being followed by an obstetrician and being of Swiss nationality are associated with knowledge of CMV. Our study showed that the education level and working in healthcare were factors associated with CMV knowledge, which were also identified by Cordier *et al.* and Jeon *et al.* [53,54].

The rate of 39% of CMV awareness found in our study is slightly higher than that reported in the literature. Jeon *et al.* found a rate of 22% in the USA, Cordier *et al.* of 34% in France, Morioka *et al.* of 18% in Japan, and Lim *et al.* of 20% in Singapore [53,54,55,56]. One possible explanation may be the high rate of enrolled women with a university degree in our study. It is probable that educated women felt more concerned and participated more frequently to the survey. The study by Jeon was performed in 2006 and was the first to reveal the serious lack of information about CMV. Thus, it could be imagined that CMV awareness has improved since then. Regarding the studies by Lim and Morioka, the fact that a screening policy was never implemented in their countries could explain this lower awareness rate. Women’s awareness about CMV ranked last with parvovirus B19, thus reflecting the results of Cordier and Lim [53,55]. Syphilis, HIV, and toxoplasmosis were known by the majority of women (>85%) as found in other studies [47,48,49,53,55]. However, these congenital infections are far less frequent than CMV and affecting between 0.04 and <1% of newborns [57,58,59,60,61,62,63,64]. In our study, CMV was more than two times less known than toxoplasmosis (39% *vs.* 87%, respectively) and yet CMV has a rate of congenital infection 15 to 50 times that of congenital toxoplasmosis [65,66]. This result is even more curious since systematic screening for toxoplasmosis has been stopped in Switzerland since 2010 [67].

Main sources of information about CMV are obstetrician, followed by media, friends, and family. Our study revealed that being followed by an obstetrician since the 1st trimester of the pregnancy increased the probability of being informed. However, less than half of the women followed by an obstetrician had been informed about CMV, thus showing a high rate of lack of information. This result is in agreement with the 44% rate shown in a prior study by Anderson [50]. Our study showed also for the first time that women followed by general practitioners or midwives were rarely informed about the risk of CMV. This reason could be related to a lack of knowledge by healthcare staff about the risks of CMV infection during pregnancy. Indeed, lack of knowledge regarding CMV infection, but also lack of time, have been reported as being associated with lack of information transmitted by healthcare providers [49,50].

At present, the only way to prevent fetal and neonatal consequences of CMV infection is through the prevention of maternal seroconversion during pregnancy by the application of strict hygiene measures [68]. Vauloup *et al.* demonstrated an 80% reduction in the rate of CMV seroconversion during pregnancy when applying correct preventive measures [41,43]. In our study, the majority of women who were aware of CMV infection also knew which preventive measures were applicable. Moreover, when CMV preventive measures were explained, most women found them easy to apply and even easier than those for the prevention of toxoplasmosis infection during pregnancy, which have already proved their efficacy [69]. These results should encourage healthcare professionals to improve information about CMV and its preventive measures.

Several studies are still ongoing to evaluate the efficacy of intravenous immunoglobulins and valacyclovir to prevent fetal and neonatal complications from CMV infection. It is crucial to find an effective treatment for fetal CMV infection, but it is still more important to prevent its occurrence. Picone *et al.* have demonstrated the efficacy of systematic information (oral and written) by showing a rate of CMV awareness of 74% [40]. As the majority of women in Geneva are followed since the 1st trimester of pregnancy, systematic oral, but also written information should be given about CMV and its preventive measures.

Our study has several limitations. The first limitation is the small sample. Another limitation is that the questionnaire was completed only by women who were willing to do so. These women may not represent the general population and there may be a bias toward educated women feeling more concerned by scientific research. This would explain also the high rate of women with a university degree. However, this selection bias could have overestimated CMV awareness and makes the need to provide more widespread information in the population even more urgent. Further studies in a much larger sample are needed to confirm the generalizability of our results.

## 5. Conclusions

Most pregnant women delivering in Geneva, Switzerland, were unaware of CMV infection, its potential risks for the fetus/newborn, and preventive measures, even though they have been correctly followed since the 1st trimester of pregnancy. Women were more frequently aware of CMV if they were followed by an obstetrician than by a midwife or a general practitioner. Although most women were followed by an obstetrician, the information rate remained low. It is crucial to improve CMV information to pregnant women from the 1st trimester in order to prevent the risks for the fetus/newborn.

## Supplementary Files

Supplementary File 1
