# Supplementary material for: Awareness of Cytomegalovirus Infection among Pregnant Women in Geneva, Switzerland: A Cross-sectional Study
Source: Int J Environ Res Public Health. 2015 Dec 2;12(12):15285–97. doi: 10.3390/ijerph121214982 (PMC4690918; doi:10.3390/ijerph121214982)
Supplement: Supplementary File 1 [file ijerph-12-14982-s001.pdf]

Supplementary Information

Awareness of Cytomegalovirus Infection among Pregnant Women in Geneva, Switzerland: A Cross-sectional Study

---

Questionnaire

Survey about the Knowledge of the Cytomegalovirus Infection (CMV)

Survey Number: _____

Please mark with a cross your answers below:

A. A few questions on various diseases:

1. **Before today, have you ever heard of**:

	Yes	No
CMV (cytomegalovirus)		
Parvovirus B 19		
Rubella		
Toxoplasmosis		
Group B Streptococcus		
Syphilis		
Hepatitis B		
HIV/AIDS		
Down Syndrom		
Syndrome alcoolo-fœtal		
Syndrome de la mort subite		
Autism		
Spina Bifida		

B. A few questions on the cytomegalovirus infection:

2. Where did you hear about the cytomegalovirus infection?

□ general practitioner (GP)
□ obstetrician at the hospital (HUG)
□ private obstetrician
□ midwife
□ paediatrician
□ From the media (TV, radio, internet, newspapers)
□ From family or friends

3. According to you, can the CMV be passed on from the mother to the foetus?

Yes □ No □ I don't know □

4. Could a newborn infected by CMV show these symptoms?

	Yes	No	I Don't Know
No danger			
Loss of hearing			
Mental retardation			

Table. *Cont.*

	Yes	No	I Don't Know
Jaundice			
Convulsions			
Microcephaly			
Congenital heart defects			
Death			

5. Which hygiene measures can prevent the CMV during the pregnancy? Choose from the list below:

	Yes	No	I Don't Know
Thorough handwashing after changing the diapers of a baby			
Not using the same cutlery as a child nor the same towel			
Using insect repellent			
Not eating raw meat or raw-milk cheese during pregnancy			
Drinking caffeine based beverages			
Using gloves to clean cat			
Not being in contact with a child's urine, tears or running nose			
Exercise during pregnancy			
Avoid kissing a child on the mouth			

6. To what extent those measures seem applicable during one's pregnancy:

	Very Easy	Fairly Easy	Fairly Hard	Very Hard
Thorough handwashing after changing the diapers of a baby				
Not using the same cutlery as a child nor the same towel				
Using insect repellent				
Not eating raw meat or raw-milk cheese during pregnancy				
Drinking caffeine based beverages				
Using gloves to clean cat				
Not being in contact with a child's urine, tears or running nose				
Exercise during pregnancy				
Avoid kissing a child on the mouth				

C. A few questions related to your pregnancy:

7. The treatment of your 1st trimester was facilitated in Geneva:

□ Yes □ No

8. Were you treated during the 1st trimester?

□ Yes □ No

9. The treatment of your 1st trimester was facilitated:

□ In town, in Geneva
□ In the Maternity Ward (HUG)

□ Outside Geneva

10. The treatment of your 1st trimester was facilitated:

□ By a general practitioner
□ By an obstetrician
□ By a midwife

11. Have you received information related to the prevention of the CMV?

Yes □ No □ I don't know □

12. Who informed you about those preventive measures?
(Please tick as many boxes apply!)

□ General Practitioner
□ Obstetrician at the Maternity Ward (HUG)
□ Private obstetrician
□ Midwife
□ Paediatrician
□ Nurse
□ Your studies
□ Media (TV, radio, internet, newspapers)
□ Family, friends

13. Have you been tested for CMV (blood test showing whether you have contracted CMV)?

Yes □ No □ I don't know □

14. When were you asked to take this test?

□ 1st trimester
□ 2nd trimester
□ 3rd trimester
□ every trimester

15. This test was:

	**Yes**	**No**	**I Don't Know**
mandatory			
proposed			
done following your request			

16. Have you received further information on preventative hygiene measures of the CMV following the test?

Yes □ No □ I don't know □

17. Following the detailed information, were you:

a. Worried:

Yes □ No □

b. Reassured:

Yes□ No □

18. Why?

D. Personal questions

19. How old are you? |___|___|
20. How many children do you have? (without the one you are currently carrying)? |___|___|
21. What is your nationality?
    ……………………………………………………………………………………
22. What is your profession?
    ……………………………………………………………………………………
23. What level of education did you reach?

□ University/Polytechnique School (EPFZ/EPFL)
□ High School diploma/ Baccalaureate
□ Apprenticeship/ Specialized formation (up to 18–19 years old)
□ Complete Compulsory School Attendance (varying depending canton/country) (up to 15–16 years old)
□ Incomplete Compulsory School Attendance (before 15–16 years old)

Thank you for bringing the completed survey to one of the researchers or in the box named <questionnaire CMV> in the midwives' office. If you forget to bring the survey back, you can also mail it to us at the address below:

© 2015 by the authors; licensee MDPI, Basel, Switzerland. This article is an open access article distributed under the terms and conditions of the Creative Commons Attribution license (http://creativecommons.org/licenses/by/4.0/).
